# DNA barcode reveals candidate species of *Scinax* and *Ololygon* (Anura: Hylidae) in Atlantic Forest

**DOI:** 10.1590/1678-4685-GMB-2021-0177

**Published:** 2022-03-07

**Authors:** Lídia Nogueira, Luís Fernando da Silva Rodrigues, Mirco Solé, Paulo Roberto Antunes de Mello Affonso, Sergio Siqueira, Iracilda Sampaio

**Affiliations:** 1 Instituto Federal de Educação, Ciência e Tecnologia, Jequié, BA, Brazil.; 2 Universidade Federal Rural da Amazônia (UFRA), Capanema, PA, Brazil.; 3 Universidade Estadual de Santa Cruz, Ilhéus, BA, Brazil.; 4 Universidade Estadual do Sudoeste da Bahia, Jequié, BA, Brazil.; 5 Universidade Federal do Pará, Instituto de Estudos Costeiros, Bragança, PA, Brazil.

**Keywords:** Biodiversity, cryptic species, mitochondrial DNA, systematic molecular, tree frogs.

## Abstract

Molecular species delimitation methods are efficient tools to identify species, including the discovery of new taxa and cryptic organisms, thus being useful to biodiversity studies. In the present work, 16S mitochondrial sequences and cytochrome oxidase I (COI) were used to evaluate the richness of species in the genus *Scinax* and *Ololygon* from a biodiversity hotspot in Atlantic Forest. A total of 109 specimens formally belonging to eight species of *Scinax* and three species of *Ololygon* were collected in 13 localities along the state of Bahia (northeastern Brazil) and one site in Espírito Santo (southeastern Brazil). Of the *Scinax* species collected in this study, three were morphologically differentiated from other described species and identified as putative new species (*Scinax* sp.1, *Scinax* sp.2 and *Scinax* sp.3). The species delimitations were inferred using three different methods: ABGD, PTP and mPTP which allowed recognizing 11 *Scinax* species and five *Ololygon* species. *Scinax* sp. 1, *Scinax* sp. 2 and *Scinax* sp. 3, have been confirmed as new putative species and *Ololygon argyreornata* possibly contains cryptic species. We suggest additional studies, including morphological and bioacoustic data to validate these new putative species.

## Introduction

Recent taxonomic revisions divided the species of the genus *Scinax* into three genera: *Ololygon* (50 spp.), including the taxa formerly recognized within the *Scinax catharinae* clade, *Scinax* (72 spp.) composed of species from *Scinax ruber* clade, and *Julianus*, placed as a sister group of *Scinax* in which *J*. *uruguayanus* and *J*. *pinima* would be synonyms of *S. uruguayanus* and *S. pinima*, respectively (Duellman et al., 2016; Frost, 2021). 

The genus *Ololygon* is widespread along the Atlantic forest from eastern Brazil southwards to northeastern Argentina and westwards into open forests of the Brazilian Cerrado (Duellman et al., 2016; Nogueira et al., 2016). The species in this genus are characterized by the lack of anterior process in suprascapula, *m. depressor mandibulae* without an origin at the dorsal fascia of the *m. dorsalis scapulae*, distal division of the middle branch of the *m. extensor digitorum comunis longus*, and insertion of this muscle at the medial side on the tendon of the *m. extensor brevis medius digiti IV* (Faivovich, 2002). The vocalization of frogs from this group is composed of pulsed notes (Hepp et al., 2017). Moreover, the karyotypes of *Ololygon* species are identified by the presence of two large submetacentric pairs while the nucleolar organizer regions (NORS) are usually located on the sixth chromosomal pair (Cardozo et al., 2011). 

The genus *Scinax* comprises small to medium-sized frogs with slightly truncate discs on fingers and toes (Duellman et al., 2016), widespread from eastern and southern Mexico to Argentina and Uruguay, Trinidad and Tobago, and St. Lucia (Frost, 2021). From a cytotaxonomic point of view, most *Scinax* species are characterized by the presence of two large metacentric pairs, and NORs on the 11^th^ pair (Cardozo et al., 2011; Nogueira et al., 2015). 

According to the IUCN (International Union for Conservation of Nature) (2021) *Scinax* species are not threatened with extinction, but many taxa lack information about distribution, population effective size and ecology. In addition, species regarded as “least concern” might encompass cryptic and range-restricted forms, as proposed for *S. alter* (Nunes et al., 2012) and *S. ruber* (Fouquet et al., 2007b). On the other hand, six species of *Ololygon* (*O. alcatraz, O. belloni, O. faivovichi*, *O*. *muriciensis*, *O. peixotoi* and *O*. *skuki*) are classified as “endangered” or “critically endangered” since they are found only on islands and/or severely deforested habitats (IUCN, 2021). Therefore, the potential presence of cryptic species related to the controversial taxonomy of both frog groups, as well as the poor information about the actual range of several taxa, indicate that alternative methods should be used to assist in their proper identification.

The rainforests of the Neotropical region harbor the largest number of undescribed amphibians, estimated in nearly 3050 species to be identified according to mathematic models (Giam et al., 2011). The incorporation of molecular tools such as DNA barcode might accelerate the recognition of new species from this region. For instance, in the Bolivian Chaco, integrative analysis revealed the presence of 69 anuran species instead of 59 as indicated in previous reports (Jansen et al., 2011). Similarly, 129 species were listed in the Amazon-Guianas using DNA barcode (Fouquet et al., 2007a), while 465 species were described in Madagascar (Vieites et al., 2009). 

In addition to identifying new species, molecular data can reveal cryptic forms, defined as genetically different but morphologically indistinguishable species (Bickford *et al*. 2007). Cryptic taxa can lead to biased estimates of regional richness and hinder conservation management policies, since putatively widespread species might actually encompass several cryptic species of restricted distribution (Funk *et al*. 2011). Amphibians usually present conserved morphological traits and, therefore, the identification of closely related species based on morphology might be difficult, while cryptic species remain overlooked (Stuart *et al*. 2006). 

In the Atlantic forest from Bahia, northeastern Brazil, 12 species of *Scinax* (*S. alter*, *S. auratus*, *S. camposseabrai*, *S. cretatus*, *S. cuspidatus*, *S. eurydice*, *S. fuscomarginatus*, *S. juncae*, *S. montivagus*, *S. nebulosus*, *S. pachycrus*, and *S. x-signatus*) and four of *Ololygon* (*O. agilis*, *O. argyreornata*, *O. catharinae*, and *O. strigilata*) have been reported (Frost, 2021). In the present study, we carried out a DNA barcode analysis based on 16S and COI genes of 11 *Scinax* species and three *Ololygon* representatives, paying particular attention to the discovery of cryptic species and identification of candidate species.

## Material and Methods

### Collection and identification of samples

**Figure 1 -:**
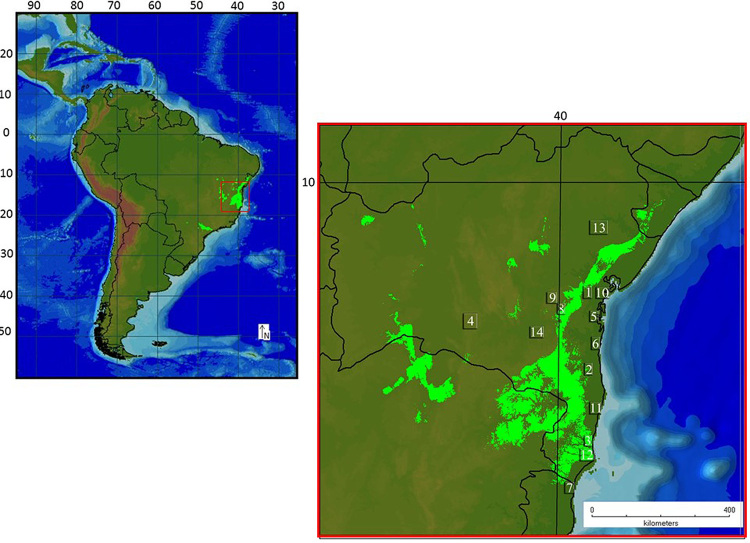
Map of collection sites of *Ololygon* and *Scinax* species: (1) Amargosa - BA, (2) Camacan - BA, (3) Caravelas - BA, (4) Guanambi - BA, (5) Igrapíuna - BA, (6) Ilhéus - BA, (7) Itaúnas - ES, (8) Jequié - BA, (9) Maracás - BA, (10) Nazaré - BA, (11) Porto Seguro - BA, (12) Prado - BA, (13) Serrinha - BA and (14) Vitória da Conquista - BA. The areas in light green indicate the putative refugia in Atlantic Forest.

A total of 109 specimens formally belonging to eight species of *Scinax* and three species of *Ololygon* were collected in Atlantic forest from 13 localities in the state of Bahia (northeastern Brazil) and one site in Espírito Santo (southeastern Brazil) (Figure 1). Most taxa were sampled in their type locality or close to it (Table S1). All specimens were identified by Dr. Mirco Solé and deposited in the Herpetological Collection of Universidade Estadual de Santa Cruz (Bahia - Brazil). Due to the absence of a taxonomic key, we identified the species seeking their description in taxonomic articles (Cruz and Peixoto 1983; Duellman and Wiens, 1992; Pombal et al., 1995; Caramaschi and Cardoso, 2006; Nunes and Pombal, 2010; Nunes *et al*., 2012; Araujo-Vieira et al., 2020). Three species of *Scinax* were morphologically identified as putative new species, being named as *Scinax* sp.1, *Scinax* sp.2, and *Scinax* sp.3 (these species were differentiated morphologically from all other species described for that region). Thus, 11 species of *Scinax* were sampled, including eight valid species and three putative new taxa (*Scinax* sp.1, *Scinax* sp.2, and *Scinax* sp.3). 

For molecular analyses, about 25 mg of muscle tissue were removed from the inner side of thighs and stored in 95% ethanol at ˗20 ^o^C.

### DNA extraction and sequencing

Total DNA was extracted using the Wizard® Genomic Purification kit (Promega) according to the manufacturer’s instructions. Two mitochondrial DNA (mtDNA) regions were amplified (16S and COI) by PCR (polymerase chain reaction) using the following primers: 16S L1 (Palumbi, 1996) + 16S H1 (Varela et al., 2007) and COI (Folmer, 1994). Cycle-sequencing was performed on both strands using BigDye Terminator v3.1 Cycle Sequencing Kit on ABI 3730 automated DNA sequencer (Applied Biosystems, Foster City, CA, USA). The chromatograms were checked using the software Finch TV Version (Geopiza, Seattle, WA), and deposited in the National Center for Biotechnology Information (NCBI) GenBank under accession numbers (Table S1).

### Data analysis

The sequences were aligned using the Clustal W tool in the software BioEdit v. 5.09 (Hall, 1999). The software GBlocks 0.91 (Castresana, 2000) was employed to remove hypervariable segments within the 16S gene, according to the following parameters: minimum number of sequences for a flank position to 71, minimum number of sequences for a flanking position to 119, maximum number of contiguous non-conserved positions to 10, minimum length of a block to 5, and half gap positions allowed. The most suitable mutation model was estimated for both COI and 16S based on Akaike Information Criteria (AIC Akaike 1974) available in the software jModel Test 0.1 (Posada, 2008). 

To increase the density of taxa, 33 sequences from GenBank were added, totaling 144 samples. These additional 16S and COI sequences were chosen based on fragment size and species identification, avoiding the utilization of undescribed or uncertain species. The species *Boana faber* was selected as outgroup. The COI sequences were translated into aminoacids and pseudogenes were absent. The data from both mitochondrial genes were concatenated in the phylogenetic analysis.

Bayesian analyses were carried out using MrBayes v.3.1.3 (Ronquist and Huelsenbeck, 2003), after concatenating 16S and COI data. The best mutation model was estimated according to Bayesian Information Criterion (BIC) in the software jModel Test 0.1 (Posada, 2008). Two runs (four chains each) with 20 million generations were performed with trees being sampled at every 1000 generations. Adequate burn-in was determined by examining likelihood scores of the heated chains for convergence on stationarity (established in 25%), as well as the effective sample size values (>200) using Tracer v. 1.5 (Rambaut and Drummond, 2007). Strongly supported relationships were considered when posterior probabilities values were equal or higher than 0.95. Additionally, a maximum likelihood (ML) tree was built using PhyML 3.0 (Guindon et al., 2010). For ML analyses, the best mutation model was estimated according to Akaike Information Criteria (AIC) in the software jModel Test 0.1 (Posada, 2008). Non-parametric bootstrapping with heuristic searches of 1000 replicates was used to estimate de confidence values of branches in ML tree. 

The nucleotide divergence for both mitochondrial genes were calculated based on Kimura-2-parameter (K2P) substitution model (Kimura, 1980), available in MEGA v. 5.0 (Tamura et al., 2011). The pairwise distances, as well as analysis of barcode gap (Meyer and Paulay, 2005) and estimation of candidate species in the dataset, were performed using the online version of Automatic Barcode Gap Discovery (ABGD) (Puillandre et al., 2012). In the ABGD, analyses were performed on the website http://wwwabi.snv.jussieu.fr/public/abgd/ using the K2P distance model. The threshold for intraspecific diversity was defined as a minimum of 0.001 and a maximum of 0.1. The default value of the barcoding gap was X¼1.5. 

For species delimitation via PTP and mPTP, we used a phylogenetic tree of maximum likelihood built in the RaxML v. 8.1 (Stamatakis, 2014), using 1oo bootstrap replications. The analyses were conducted in the PTP and mPTP web servers (http://species.h-its.org/ and http://mptp.h-its.org/#/tree).

## Results

The final dataset included 990 base pairs of mitochondrial genes (629 pb COI and 384 pb 16S) for 144 specimens, morphologically associated with 16 species of *Scinax* and four species of *Ololygon*.

**Figure 2 -:**
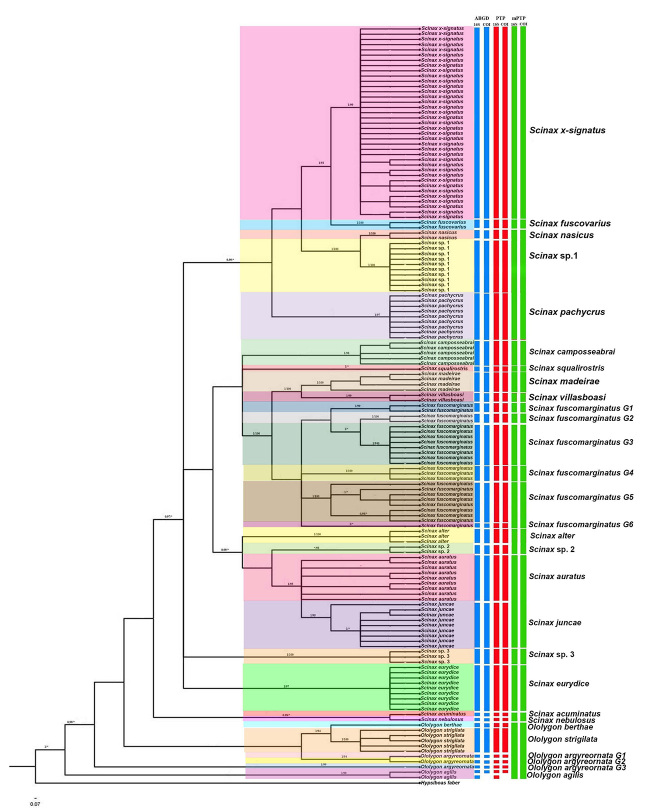
Bayesian inference (BI) topology with posterior probability values (pp > 95%) and maximum likelihood (ML) bootstrap support (>70%) based on concatenated analysis of 16S and COI genes (990 bp) in *Scinax* and *Ololygon* species. Vertical bars correspond to each lineage considered as a potential species. The delimitation species were inferred according to ABGD, PTP and mPTP methods.

Monophyletic groups were recovered in phylogenetic inferences with strong statistical support ( > 0.98/94), most of them equivalent to the morphologically identified species (*O. agilis, O. strigilata, Scinax alter, Scinax auratus, Scinax camposseabrai, Scinax eurydice, Scinax fuscomarginatus, Scinax juncae, Scinax pachycrus, Scinax* sp.1*, Scinax* sp.2*, Scinax* sp.3, and *Scinax x-signatus*) (Figure 2). 

The samples of *Scinax x-signatus* were reliably clustered in a single clade, being closely related to *S*. *fuscovarius* (Figure 2). The nucleotide divergence between both sister taxa was equal to 8% for the 16S and 17% for COI (Table 1). 

**Table 1 -:** Interspecific nucleotide divergence in COI (above diagonal) and 16S (below diagonal) within *Scinax* based on K2P model.

	1	2	3	4	5	6	7	8	9	10	11	12	13	14	15	16	17	18	19	20	21	22	23	24	25	26	27	28	29
1		0.11	0.11	0.10	0.11	0.13	0.11	0.14	0.12	0.14	0.15	0.14	0.13	0.13	0.16	0.14	0.14	0.15	0.11	0.15	0.14	0.12	0.12	0.15	0.13	0.14	0.11	0.16	0.17
2	0.26		0.11	0.09	0.08	0.09	0.10	0.13	0.11	0.12	0.12	0.12	0.12	0.12	0.14	0.13	0.12	0.15	0.11	0.14	0.15	0.11	0.13	0.16	0.10	0.13	0.11	0.13	0.15
3	0.25	0.23		0.06	0.08	0.09	0.08	0.12	0.08	0.10	0.11	0.13	0.13	0.14	0.15	0.13	0.13	0.12	0.08	0.14	0.13	0.10	0.12	0.12	0.09	0.10	0.07	0.15	0.16
4	0.26	0.28	0.18		0.08	0.08	0.09	0.11	0.07	0.11	0.11	0.14	0.15	0.13	0.17	0.13	0.13	0.14	0.05	0.14	0.15	0.10	0.11	0.15	0.10	0.13	0.08	0.14	0.17
5	0.26	0.22	0.25	0.22		0.08	0.11	0.14	0.11	0.11	0.13	0.13	0.13	0.13	0.15	0.12	0.12	0.13	0.10	0.13	0.15	0.13	0.13	0.15	0.12	0.12	0.10	0.13	0.16
6	0.24	0.27	0.26	0.20	0.20		0.11	0.15	0.11	0.11	0.13	0.12	0.13	0.11	0.15	0.13	0.13	0.15	0.09	0.11	0.16	0.11	0.12	0.16	0.12	0.14	0.10	0.12	0.18
7	0.27	0.29	0.23	0.19	0.24	0.22		0.06	0.06	0.09	0.09	0.09	0.11	0.11	0.12	0.11	0.11	0.10	0.08	0.11	0.13	0.07	0.11	0.11	0.06	0.10	0.03	0.12	0.15
8	0.25	0.28	0.26	0.23	0.25	0.25	0.26		0.07	0.13	0.09	0.13	0.14	0.12	0.15	0.15	0.12	0.12	0.09	0.15	0.15	0.09	0.12	0.12	0.05	0.10	0.06	0.15	0.17
9	0.26	0.27	0.24	0.21	0.26	0.23	0.23	0.19		0.09	0.08	0.12	0.12	0.10	0.13	0.11	0.10	0.11	0.03	0.11	0.11	0.07	0.09	0.10	0.05	0.08	0.04	0.12	0.15
10	0.27	0.27	0.24	0.22	0.20	0.24	0.21	0.26	0.28		0.11	0.15	0.15	0.13	0.16	0.13	0.13	0.12	0.10	0.14	0.14	0.11	0.10	0.12	0.11	0.13	0.09	0.15	0.13
11	0.24	0.27	0.25	0.21	0.24	0.23	0.20	0.22	0.25	0.25		0.11	0.11	0.11	0.13	0.12	0.11	0.11	0.09	0.15	0.12	0.11	0.09	0.09	0.08	0.09	0.09	0.13	0.14
12	0.24	0.25	0.25	0.24	0.25	0.28	0.27	0.23	0.25	0.25	0.21		0.04	0.05	0.05	0.05	0.05	0.12	0.13	0.09	0.13	0.13	0.12	0.12	0.10	0.14	0.11	0.08	0.16
13	0.25	0.27	0.25	0.24	0.23	0.27	0.28	0.25	0.25	0.25	0.24	0.13		0.04	0.06	0.06	0.05	0.12	0.13	0.07	0.12	0.13	0.12	0.12	0.10	0.12	0.11	0.08	0.15
14	0.29	0.27	0.29	0.26	0.27	0.29	0.25	0.24	0.26	0.28	0.22	0.13	0.13		0.07	0.05	0.05	0.12	0.11	0.08	0.13	0.13	0.11	0.13	0.10	0.12	0.10	0.07	0.16
15	0.25	0.26	0.28	0.20	0.25	0.25	0.26	0.24	0.22	0.25	0.21	0.14	0.11	0.15		0.04	0.06	0.14	0.14	0.11	0.15	0.15	0.13	0.14	0.11	0.14	0.12	0.10	0.15
16	0.26	0.26	0.27	0.20	0.24	0.23	0.24	0.21	0.22	0.25	0.21	0.15	0.14	0.14	0.11		0.04	0.12	0.12	0.10	0.14	0.14	0.12	0.13	0.11	0.14	0.10	0.08	0.14
17	0.27	0.27	0.28	0.22	0.25	0.24	0.24	0.23	0.23	0.27	0.23	0.17	0.15	0.14	0.13	0.07		0.14	0.10	0.10	0.16	0.13	0.13	0.14	0.09	0.13	0.09	0.09	0.15
18	0.26	0.25	0.24	0.19	0.21	0.24	0.23	0.23	0.23	0.24	0.20	0.25	0.28	0.26	0.25	0.25	0.25		0.13	0.14	0.11	0.10	0.09	0.10	0.12	0.08	0.10	0.13	0.08
19	0.25	0.29	0.27	0.24	0.27	0.26	0.27	0.19	0.10	0.27	0.24	0.23	0.23	0.25	0.22	0.22	0.22	0.24		0.13	0.13	0.09	0.09	0.12	0.06	0.10	0.06	0.12	0.15
20	0.27	0.27	0.27	0.26	0.24	0.25	0.27	0.22	0.26	0.26	0.20	0.20	0.20	0.20	0.18	0.20	0.21	0.27	0.25		0.16	0.12	0.15	0.15	0.13	0.15	0.11	0.08	0.19
21	0.23	0.26	0.24	0.21	0.20	0.24	0.26	0.22	0.26	0.28	0.23	0.22	0.22	0.24	0.21	0.23	0.24	0.23	0.24	0.23		0.13	0.07	0.05	0.13	0.09	0.12	0.16	0.12
22	0.28	0.27	0.27	0.25	0.27	0.27	0.24	0.28	0.24	0.30	0.26	0.28	0.28	0.30	0.27	0.26	0.28	0.29	0.24	0.29	0.27		0.11	0.13	0.09	0.11	0.08	0.13	0.15
23	0.24	0.26	0.25	0.21	0.21	0.21	0.26	0.22	0.25	0.23	0.19	0.24	0.24	0.26	0.21	0.23	0.24	0.18	0.26	0.24	0.21	0.26		0.08	0.10	0.08	0.10	0.16	0.13
24	0.25	0.27	0.22	0.23	0.23	0.24	0.23	0.25	0.24	0.27	0.22	0.24	0.24	0.24	0.23	0.24	0.24	0.22	0.23	0.28	0.17	0.28	0.22		0.12	0.08	0.10	0.15	0.12
25	0.26	0.28	0.25	0.24	0.23	0.25	0.25	0.21	0.17	0.27	0.23	0.25	0.28	0.26	0.24	0.23	0.24	0.21	0.17	0.26	0.24	0.26	0.22	0.23		0.09	0.04	0.13	0.17
26	0.26	0.26	0.25	0.21	0.24	0.25	0.23	0.21	0.21	0.24	0.22	0.24	0.22	0.23	0.22	0.20	0.21	0.24	0.23	0.25	0.20	0.27	0.25	0.22	0.24		0.08	0.15	0.11
27	0.25	0.28	0.27	0.25	0.26	0.26	0.26	0.19	0.21	0.25	0.21	0.22	0.22	0.24	0.25	0.24	0.24	0.24	0.24	0.24	0.24	0.25	0.24	0.24	0.24	0.23		0.12	0.15
28	0.30	0.28	0.28	0.27	0.23	0.25	0.28	0.25	0.27	0.26	0.24	0.19	0.19	0.22	0.21	0.20	0.19	0.26	0.24	0.19	0.24	0.33	0.25	0.26	0.27	0.26	0.27		0.16
29	0.26	0.27	0.25	0.19	0.18	0.22	0.21	0.23	0.25	0.25	0.21	0.24	0.25	0.24	0.25	0.23	0.24	0.17	0.23	0.26	0.22	0.24	0.21	0.22	0.21	0.22	0.24	0.24	

Based on morphological traits, three putative new species were suggested in the present study, being named as *Scinax* sp.1, *Scinax* sp. 2, and *Scinax* sp. 3. The phylogenetic inferences, ABGD and PTP confirmed this suggestion, discriminating these groups into three distinct clusters with high statistical support (Figure 2). The minimum and maximum values of nucleotide divergence for these three taxa in relation to other *Scinax* species were: *Scinax* sp. 1: 5% ˗ 15% and 17% ˗ 27%; *Scinax* sp. 2: 4.5% ˗ 13% and 17 ˗ 28%; and *Scinax* sp. 3: 7.5% ˗ 14% and 20% ˗ 26% for the 16S and COI genes, respectively (Table 1). The mPTP method recognized only *Scinax* sp.3, while *Scinax* sp.1 and *Scinax* sp. 2 were grouped with related species (*Scinax* sp.1- *S*. *nasicus* and *S*. *pachycrus* and *Scinax* sp. 2 - *S*. *alter*) (Figure 2).

The DNA sequences of *S*. *fuscomarginatus* from the present study (samples collected in Caravelas, southern Bahia, northeastern Brazil) were compared to those available in GenBank and they grouped with specimens from distinct regions in central, southeastern and northeastern Brazil. Indeed, several clusters were observed within *S*. *fuscomarginatus*, with divergence values higher than 4.0 % and 7.0 % for 16S and COI fragments, respectively (Table 1). The species delimitation methods used in this study confirm the separation of the species *S*. *fucomarginatus* in at least six species groups (Figure 2). On the other hand, *S*. *madeirae* and *S*. *villasboasi* were closely related to *S*. *fuscomarginatus*.

The cladogram revealed a phylogenetic proximity between *S*. *alter*, *S*. *auratus*, *S*. *juncae*, and *Scinax* sp. 2, with divergence values ranging from 3.5 to 9% (16S) and 10 to 19% (COI) (Figure 2, Table 1).

Surprisingly, the three specimens identified as *O*. *argyreornata*, collected in Porto Seguro (2) and Ilhéus (1), about 300 km apart in the southern coast of Bahia, presented high nucleotide divergence among each other and in relation to all other *Scinax* species (mean value of 8.7 ± 2.5% for the 16S, and 23.0 ± 5% for the COI) (Table 1). Therefore, the genetic differentiation within *O*. *argyreornata* suggests that these specimens should represent distinct taxonomic units. 

The species delimitation methods, ABGD and PPT were coincident in the recognition of the species of *Scinax* and *Ololygon*, resulting in 23 and 7 evolutionary units, respectively. The mPTP was unable to distinguish some pairs of sister species that are easily recognizable based on morphology (*S*. *pachycrus* x *S*. *nasicus* x *Scinax* sp.1 / *S*. *alter* x *Scinax* sp. 2 and *S*. *auratus* x *S*. *juncae*). For the genus *Ololygon*, the mPTP method failed in distinguishing different species (Figure 2). 

## Discussion

In the present study, species delimitation was inferred based on distinct approaches, distance matrix and ABGD, PTP and mPTP algorithms. These tools complemented the morphological identification and indicated populations that need further revisions from an integrative perspective, particularly in relation to *O*. *argyneornatus*. 

Specimens of this nominal taxon from two localities (~ 300 km apart) in southern Bahia were divided into three distinct lineages. This pattern could be related to biogeographic events in Atlantic forest, particularly related to the formation of refugia in Pleistocene. During this period, climatic changes, such as glaciation reduced large forest areas into patched fragments (forest refugia), thus, isolating individuals into small populations. Putatively, after warming, the habitats expanded once again leading to a secondary contact of populations (Carnaval et al., 2009). According to this model, the interruption of gene flow during isolation in refugia would determine genetic diversification of lineages and eventually reproductive isolation. 

The genetic analyses also recognized *S*. *fuscomarginatus* as a species complex in spite of the lack of significant differences in morphometric and advertisement call data (Brusqueti *et al*., 2014). Therefore, the nominal species *S. fuscomarginatus* actually encompasses cryptic species, i.e. genetically and reproductively isolated species that lack morphological differentiation (Bickford et al., 2007). 

Cryptic species are particularly common in anurans once their mating success relies more on acoustic patterns and pheromones than on visual signs (Bickford et al., 2007). Therefore, pre-zygotic barriers rather evolve from changes in other traits but morphological traits. Cytogenetic data support the discrimination between *S*. *trilineatus* and *S*. *fuscomarginatus* (Nogueira et al., 2015), in spite of their high similarity in morphological and vocalization traits (Brusquetti et al., 2014). Studies focusing on gene flow are highly encouraged in these *Scinax* groups to confirm the present inferences, like those performed by Gehara et al. (2014). 

The present genetic data also confirmed the taxonomic status of *S*. *camposseabrai*. This species was formerly regarded as a subspecies of *S*. *x-signatus*, until it was revalidated by Caramaschi and Cardoso (2006). This taxon is found in decidual and semidecidual areas of Atlantic forest in Bahia (northeastern Brazil) and Minas Gerais (southeastern Brazil), being characterized by explosive spawning during short periods of rainfall (Cândido et al., 2012). 

On the other hand, the samples of *S*. *x-signatus* from several localities formed a single cluster (Figure 2). It should be pointed out that specimens from this clade present a remarkable variation in coloration, texture and pattern of patches in skin, particularly noticeable in populations from the state of Bahia. Once this represents a monophyletic group, the external morphological differences are likely to represent local adaptive traits related to environmental differences throughout their range.

Differently from *S*. *x-signatus*, the species *S. auratus* has a more restricted distribution on rocky formation in inner portions or open areas in fragment borders of Atlantic forest along northeastern Brazil (Alves et al., 2004). This species, based on biological and morphological traits, is closely related to *Scinax alter, S. cretatus, S. crospedospilus*, *S. cuspidatus*, *S. imbegue, S. juncae* and *S. tymbamirim* (Pombal et al., 1995; Alves *et al*., 2004; Nunes and Pombal, 2010; Nunes, 2011, 2012; Mercês and Juncá, 2012). The molecular identification supported this inference, showing that *S*. *auratus* is genetically related to *S*. *juncae*, *S*. *alter,* as well as to *Scinax* sp. 2 (Figure 2). 

Furthermore, all new *Scinax* taxa indicated in the present study were collected in putative biodiversity refugia in Atlantic forest (Figure 1). Based on the nomenclature proposed by Padial et al. (2010), *Scinax* sp.1, *Scinax* sp. 2, *Scinax* sp. 3, *O. argyreornata* 1, *O. argyreornata* 2, and *O. argyreornata* 3 should be categorized as unconfirmed candidate species (UCS) that should be further analyzed to reach a valid taxonomic status.

Our study emphasizes the importance of incorporating distinct methods to species identification, as recommended in integrative taxonomy (Dayrat, 2005; Padial et al., 2010). Accordingly, the molecular data identified 11 lineages of *Scinax* and 5 of *Ololygon*, including five new candidate species, thus contributing to the knowledge about the richness of anurans in Atlantic forest and definition of priority areas for biodiversity conservation.

## Supplementary material

The following online material is available for this article:

Table S1 - Description of the samples represented in this study.
