# Supplementary material for: DNA barcode reveals candidate species of Scinax and Ololygon (Anura: Hylidae) in Atlantic Forest
Source: Genet Mol Biol. 2022 Mar 7;45(1):e20210177. doi: 10.1590/1678-4685-GMB-2021-0177 (PMC8905458; doi:10.1590/1678-4685-GMB-2021-0177)
Supplement: Table S1 - [file 1415-4757-GMB-45-1-e20210177-s1.pdf]

Genetics and Molecular Biology
https://doi.org/10.1590/1678-4685-GMB-2021-0177

Supplementary Material to "DNA barcode reveals candidate species of *Scinax* and *Ololygon* (Anura: Hylidae) in Atlantic Forest"

Table S1 - Description of the samples: Number of voucher, species, type locality, sampled location, geographic coordinates, number of access in GenBank and reference.

Voucher	Species	Type locality	Location sampled	Geografic Coordinates	Number of acess in GenBank 16S	COI	Reference
	Boana faber		**	**	JQ627206.1	KM024342.1	(Unpublished)
IIBPH277	*S. acuminatus*		San Jose, Neembucu, Paraguay	26°39'00"S, 57°56'00"W	AY843753.1	KJ004281.1	Brusquetti *et al.* 2014
UESC9759	*O. agilis*	Linhares, Espirito Santo, Brazil	Itaúnas, Espirito Santo, Brazil	18°25'16"S, 39°42'33"W	OK161102	OK157346	This study
UESC9814	*O. agilis*		Porto Seguro, Bahia, Brazil	16°27'4"S, 39°3'53"W	OK161103		This study
288*	*S. alter*	S. Leopoldina, Espirito Santo, Brazil	Itaúnas, Espirito Santo, Brazil	18°25'16"S, 39°42'33"W	OK161112	OK157347	This study
289*	*S. alter*		Itaúnas, Espirito Santo, Brazil	18°25'16"S, 39°42'33"W	OK161113		This study
290*	*S. alter*		Caravelas, Bahia, Brazil	17°42' 49"S, 39°14'54"W	OK161114		This study
495*	*O. argyreornata*	Rio Mutum, Espirito Santo, Brazil	Ilhéus, Bahia, Brazil	14°47'50"S, 39°2' 8"W	OK161110	OK157343	This study
UESC9811	*O. argyreornata*		Porto Seguro, Bahia, Brazil	16°27'4"S, 39°3'53"W	OK161109	OK157341	This study
UESC9812	*O. argyreornata*		Porto Seguro, Bahia, Brazil	16°27'4"S, 39°3'53"W	OK161111	OK157342	This study
492*	*S. auratus*	Santa Inês, Bahia, Brazil	Nazaré, Bahia, Brazil	13°2'8"S, 39°0'12"W	OK161122		This study
UESC11148	*S. auratus*		Nazaré, Bahia, Brazil	13°2'8"S, 39°0'12"W	OK161123		This study
139*	*S. auratus*		Jequié, Bahia, Brazil	13°51'4"S, 40°4'52"W	OK161116	OK157348	This study
UESC11051	*S. auratus*		Jequié, Bahia, Brazil	13°51'4"S, 40°4'52"W	OK161115		This study
UESC11052	*S. auratus*		Jequié, Bahia, Brazil	13°51'4"S, 40°4'52"W	OK161117		This study
UESC11053	*S. auratus*		Jequié, Bahia, Brazil	13°51'4"S, 40°4'52"W	OK161118		This study
UESC11054	*S. auratus*		Jequié, Bahia, Brazil	13°51'4"S, 40°4'52"W	OK161121		This study
UESC11055	*S. auratus*		Jequié, Bahia, Brazil	13°51'4"S, 40°4'52"W	OK161119		This study
UESC11056	*S. auratus*		Jequié, Bahia, Brazil	13°51'4"S, 40°4'52"W	OK161120		This study
IIBPH1396	*O. berthae*		Alto Vera, Itapua, Paraguay	26°35'00"S, 55°34'00"W	KJ004191.1	KJ004284.1	Brusquetti *et al.* 2014
UESC11020	*S. camposseabrai*	Maracás, Bahia, Brazil	Maracás, Bahia, Brazil	13°25'42"S, 40°26'16"W	OK161133		This study
UESC11021	*S. camposseabrai*		Maracás, Bahia, Brazil	13°25'42"S, 40°26'16"W	OK161134		This study
UESC11022	*S. camposseabrai*		Maracás, Bahia, Brazil	13°25'42"S, 40°26'16"W	OK161135		This study
UESC11023	*S. camposseabrai*		Maracás, Bahia, Brazil	13°25'42"S, 40°26'16"W	OK161137		This study
UESC11026	*S. camposseabrai*		Maracás, Bahia, Brazil	13°25'42"S, 40°26'16"W	OK161136		This study
UESC11005	*S. eurydice*	Maracás, Bahia, Brazil	Jequié,Bahia,Brazil	13° 51' 4" S, 40°4'52"W	OK161150		This study
UESC11007	*S. eurydice*		Jequié,Bahia,Brazil	13° 51' 4" S, 40°4'52"W	OK161151	OK157339	This study

Voucher	Specie	Type locality	Location sampled	Geografic Coordinates	Number of acess in GenBank		Reference
					16S	COI	
UESC11049	*S. eurydice*		Jequié,Bahia,Brazil	13° 51′ 4″ S, 40°4′52″W	OK161149		This study
UESC11050	*S. eurydice*		Jequié,Bahia,Brazil	13° 51′ 4″ S, 40°4′52″W	OK161152		This study
UESC11047	*S. eurydice*		Ilhéus,Bahia,Brazil	14°47′ 50″S, 39°2′8″W	OK161148		This study
311*	*S. eurydice*		Ilhéus,Bahia,Brazil	14°47′ 50″S, 39°2′8″W	OK161155		This study
UESC11138	*S. eurydice*		Jequié,Bahia,Brazil	13° 51′ 4″ S, 40°4′52″W	OK161153	OK157340	This study
UESC11139	*S. eurydice*		Jequié,Bahia,Brazil	13° 51′ 4″ S, 40°4′52″W	OK161154		This study
CFBH29521	*S. fuscomarginatus*	Belo Horizonte,Minas Gerais , Brazil	Boa Vista, Roraima, Brazil	02°49'12" N, 60°40'23"W	KJ004121.1	KJ004216.1	Brusquetti *et al.* 2014
CAB5751	*S. fuscomarginatus*		Santa Elena de Uairen, Bolivar Venezuela	04°37′00″N, 61°08′00″W	KJ004120.1	KJ004192.1	Brusquetti *et al.* 2014
CHUNB51003	*S. fuscomarginatus*		Luis Eduardo Magalhaes, Bahia, Brazil	12°5′58″S, 45°47′54″W	KJ004123.1	KJ004233.1	Brusquetti *et al.* 2014
CHUNB38023	*S. fuscomarginatus*		Parana, Tocantins, Brazil	12°36′55″S, 47°52′59″W	KJ004122.1	KJ004228.1	Brusquetti *et al.* 2014
CFBH20548	*S. fuscomarginatus*		Jaborandi, Bahia, Brazil	13°37′10″S, 44°25′58″W	KJ004126.1	KJ004205.1	Brusquetti *et al.* 2014
CHUNB36465	*S. fuscomarginatus*		Sao Domingos, Goias, Brazil	13°16′00″S, 46°44′00″W	KJ004128.1	KJ004227.1	Brusquetti *et al.* 2014
CFBH24356	*S. fuscomarginatus*		Lagoa Santa, Minas Gerais, Brazil	19°37′38″S, 43°53′23″W	KJ004132.1	KJ004211.1	Brusquetti *et al.* 2014
CFBH23345	*S. fuscomarginatus*		Caetite, Bahia, Brazil	14°04′10″S, 42°28′30″W	KJ004131.1	KJ004210.1	Brusquetti *et al.* 2014
MJ1355	*S. fuscomarginatus*		Santa Cruz, San Sebastian, Bolivia	16.35963S, 62.00005W	JF790008.1		Jansen *et al.* 2011
MJ1288	*S. fuscomarginatus*		Santa Cruz, San Matias, Bolivia	17.03272 S 58.53448 W	JF789995.1		Jansen *et al.* 2011
MNKA9624	*S. fuscomarginatus*		Caparu, Santa Cruz, Bolivia	12°17′00″S, 66°15′00″W	KJ004114.1	KJ004257.1	Brusquetti *et al.* 2014
CFBH10248	*S. fuscomarginatus*		Aguaracema, Tocantins, Brazil	8° 48′ 31″ S, 49°33′28″W	KJ004147.1	KJ004195.1	Brusquetti *et al.* 2014
CFBH18678	*S. fuscomarginatus*		São Paulo, São Paulo, Brazil	22°31′57″S, 52°10′03″W	KJ004111.1	KJ004201.1	Brusquetti *et al.* 2014
CFBH11433	*S. fuscomarginatus*		Araguaina, Tocantins, Brazil	07°11′28″S, 48°12′26″W	KJ004151.1	KJ004198.1	Brusquetti *et al.* 2014
ZUFG5467	*S. fuscomarginatus*		Jatai, Goias, Brazil	13°50′00″S, 46°26′00″W	KJ004171.1	KJ004266.1	Brusquetti *et al.* 2014
ZUFG5466	*S. fuscomarginatus*		Jatai, Goias, Brazil	13°50′00″S, 46°26′00″W	KJ004170.1	KJ004265.1	Brusquetti *et al.* 2014
ZUFG5465	*S. fuscomarginatus*		Jatai, Goias, Brazil	13°50′00″S, 46°26′00″W	KJ004169.1	KJ004264.1	Brusquetti *et al.* 2014
CTMZ6684	*S. fuscomarginatus*		Paranaita, Mato Grosso, Brazil	09°39′53″S, 56°28′36″W	KJ004173.1	KJ004242.1	Brusquetti *et al.* 2014
CTMZ6223	*S. fuscomarginatus*		Itauba, Mato Grosso, Brazil	11°03′42″S, 55°16′35″W	KJ004165.1	KJ004241.1	Brusquetti *et al.* 2014
CTMZ6194	*S. fuscomarginatus*		Colider, Mato Grosso, Brazil	10°49′04″S, 55°27′03″W	KJ004164.1	KJ004240.1	Brusquetti *et al.* 2014
UESC9790	*S. fuscomarginatus*		Caravelas ,Bahia,Brazil	17°42′49″S, 39°14′54″W	OK161170	OK157361	This study
UESC9788	*S. fuscomarginatus*		Caravelas ,Bahia,Brazil	17°42′49″S, 39°14′54″W	OK161171		This study
UESC9791	*S. fuscomarginatus*		Caravelas ,Bahia,Brazil	17°42′49″S, 39°14′54″W	OK161172		This study
UESC9789	*S. fuscomarginatus*		Caravelas ,Bahia,Brazil	17°42′49″S, 39°14′54″W	OK161173		This study

Voucher	Specie	Type locality	Location sampled	Geografic Coordinates	Number of acess in GenBank		Reference
					16S	COI	
MNKA9772	*S. fuscovarius*		**	**	JF790014.1	JQ627327.1	(Unpublished)
MNKA9695	*S. fuscovarius*		**	**	JF790013.1	JQ627323.1	(Unpublished)
528*	*S. juncae*	Igrapiúna, Bahia, Brazil	Igrapiúna, Bahia, Brazil	13°43'57''S, 39°9'2''W	OK161126		This study
522*	*S. juncae*		Porto Seguro, Bahia, Brazil	16°27'4"S, 39°3'53"W	OK161124	OK157362	This study
530*	*S. juncae*		Camacan, Bahia, Brazil	15°24'51"S, 39°30'4"W	OK161127	OK157363	This study
531*	*S. juncae*		Igrapiúna, Bahia, Brazil	13°43'57''S, 39°9'2''W	OK161128		This study
532*	*S. juncae*		Igrapiúna, Bahia, Brazil	13°43'57''S, 39°9'2''W	OK161129		This study
536*	*S. juncae*		Camacan, Bahia, Brazil	15°24'51"S, 39°30'4"W	OK161132		This study
527*	*S. juncae*		Camacan, Bahia, Brazil	15°24'51"S, 39°30'4"W	OK161125		This study
535*	*S. juncae*		Igrapiúna, Bahia, Brazil	13°43'57''S, 39°9'2''W	OK161131	OK157364	This study
534*	*S. juncae*		Igrapiúna, Bahia, Brazil	13°43'57''S, 39°9'2''W	OK161130		This study
MNKA9445	*S.madeirae*		San Sebastian, Santa Cruz, Bolivia	16°21'00"S, 58°41'00"W	KJ004099.1	KJ004278.1	Brusquetti *et al.* 2014
SMF88220	*S.madeirae*		Caparu, Santa Cruz, Bolivia	12°17'00"S, 66°15'00"W	KJ004102.1	KJ004279.1	Brusquetti *et al.* 2014
CFBH25469	*S.madeirae*		Porto Velho, Rondonia, Brazil	08°45'43"S, 63°54'14"W	KJ004277.1	KJ004101.1	Brusquetti *et al.* 2014
MNKA9647	*S. nasicus*		Bolivia	14.9121S, 61.0825 W	KF723109.1		Schulze *et al.* 2013
IIBPH262	*S. nasicus*		Estancia San Jose, Neembucu, Paraguay	26°39'00"S, 57°56'00"W	KJ004188.1	KJ004280.1	Brusquetti *et al.* 2014
CFBHT10951	*S. nebulosus*		Baixa Grande, Piaui, Brazil	05°59'00"S, 42°57'00"W	KJ004190.1	KJ004282.1	Brusquetti *et al.* 2014
UESC11027	*S. pachycrus*	Poção, Pernambuco, Brazil	Maracás, Bahia, Brazil	13°25'42"S, 40°26'16"W	OK161140	OK157349	This study
UESC11002	*S. pachycrus*		Jequié, Bahia, Brazil	13°51'4"S, 40°4'52"W	OK161139		This study
UESC11008	*S. pachycrus*		Jequié, Bahia, Brazil	13°51'4"S, 40°4'52"W	OK161141		This study
UESC11009	*S. pachycrus*		Jequié, Bahia, Brazil	13°51'4"S, 40°4'52"W	OK161142		This study
UESC11036	*S. pachycrus*		Jequié, Bahia, Brazil	13°51'4"S, 40°4'52"W	OK161144		This study
UESC11037	*S. pachycrus*		Jequié, Bahia, Brazil	13°51'4"S, 40°4'52"W	OK161147		This study
UESC11048	*S. pachycrus*		Jequié, Bahia, Brazil	13°51'4"S, 40°4'52"W	OK161143		This study
217*	*S. pachycrus*		Vitória da Conquista, Bahia, Brazil	14° 51' 53"S, 40°50'13"W	OK161145		This study
218*	*S. pachycrus*		Vitória da Conquista, Bahia, Brazil	14°51'53"S, 40°50'13"W	OK161146		This study
UESC9798	*S.* sp. 1		Prado, Bahia, Brazil	17°19'57"S, 39°13' 51"W	OK161156	OK157350	This study
UESC9796	*S.* sp. 1		Caravelas, Bahia, Brazil	17°42'49" S, 39°14'54"W	OK161157		This study
UESC9794	*S.* sp. 1		Caravelas, Bahia, Brazil	17°42'49" S, 39°14'54"W	OK161158	OK157351	This study
UESC9795	*S.* sp. 1		Caravelas, Bahia, Brazil	17°42'49" S, 39°14'54"W	OK161159		This study
UESC9797	*S.* sp. 1		Caravelas, Bahia, Brazil	17°42'49" S, 39°14'54"W	OK161160	OK157358	This study
314*	*S.* sp. 1		Ilhéus, Bahia, Brazil	14°47'50"S, 39°2'8"W	OK161163		This study
306*	*S.* sp. 1		Ilhéus, Bahia, Brazil	14°47'50"S, 39°2'8"W	OK161162	OK157353	This study

Voucher	Specie	Type locality	Location sampled	Geografic Coordinates	Number of acess in GenBank		Reference
					16S	COI	
308*	*S.* sp. 1		Ilhéus, Bahia, Brazil	14°47'50"S, 39°2'8"W	OK161164	OK157355	This study
309	*S.* sp. 1		Ilhéus, Bahia, Brazil	14°47'50"S, 39°2'8"W		OK157356	This study
310*	*S.* sp. 1		Ilhéus, Bahia, Brazil	14°47'50"S, 39°2'8"W	OK161165	OK157357	This study
304*	*S.* sp.1		Ilhéus, Bahia, Brazil	14°47' 50"S, 39°2'8"W	OK161161	OK157352	This study
UESC11147	S. sp. 2		Ilhéus, Bahia, Brazil	14°47'50"S, 39°2'8"W	OK161167	OK157359	This study
422*	*S.* sp. 2		Ilhéus, Bahia, Brazil	14°47'50"S, 39°2'8"W	OK161166		This study
UESC11034	*S.* sp. 3		Jequié, Bahia, Brazil	13°51'4"S, 40°4'52"W	OK161138	OK157360	This study
305*	*S.* sp. 3		Ilhéus, Bahia, Brazil	14°47'50"S, 39°2'8"W	OK161168		This study
312*	*S.* sp. 3		Ilhéus, Bahia, Brazil	14°47'50"S, 39°2'8"W	OK161169		This study
CFBH21975	*S.squalirostris*		Serra da Bocaina, São Paulo, Brazil	22°38'42"S, 44°34'40"W	KJ004187.1	KJ004283.1	Brusquetti *et al.* 2014
UESC11077	*O. strigilata*	Ibirapitanga, Bahia, Brazil	Camacan, Bahia, Brazil	15°24'51"S, 39°30' 4"W	OK161104		This study
UESC11079	*O. strigilata*		Camacan, Bahia, Brazil	15°24'51"S, 39°30' 4"W	OK161105	OK157344	This study
UESC11080	*O. strigilata*		Camacan, Bahia, Brazil	15°24'51"S, 39°30' 4"W	OK161106		This study
313*	*O. strigilata*		Itaúnas, Espirito Santo, Brazil	18°25'16"S 39°42'33"W	OK161107		This study
494*	*O. strigilata*		Amargosa, Bahia, Brazil	13°1'39" S, 39°36'23"W	OK161108	OK157345	This study
CHUNB34502	*S. villasboasi*		Serra do Cachimbo, Pará, Brazil	07°08'52"S, 55°22'52"W	KJ004103.1	KJ004269.1	Brusquetti *et al.* 2014
CHUNB34507	*S. villasboasi*		Serra do Cachimbo, Pará, Brazil	07°08'52"S, 55°22'52"W	KJ004105.1	KJ004271.1	Brusquetti *et al.* 2014
UESC11010	*S. x-signatus*	Bahia*, Brazil	Jequié, Bahia, Brazil	13°51'4"S, 40°4'52"W	OK161182	OK157312	This study
UESC11014	*S. x-signatus*		Jequié, Bahia, Brazil	13°51'4"S, 40°4'52"W	OK161184	OK157313	This study
UESC11015	*S. x-signatus*		Jequié, Bahia, Brazil	13°51'4"S, 40°4'52"W	OK161185	OK157314	This study
UESC11016	*S. x-signatus*		Jequié, Bahia, Brazil	13°51'4"S, 40°4'52"W	OK161186	OK157315	This study
UESC11018	*S. x-signatus*		Jequié, Bahia, Brazil	13°51'4"S, 40°4'52"W	OK161187	OK157316	This study
UESC11001	*S. x-signatus*		Jequié, Bahia, Brazil	13°51'4"S, 40°4'52"W	OK161188	OK157317	This study
UESC11013	*S. x-signatus*		Jequié, Bahia, Brazil	13°51'4"S, 40°4'52"W	OK161189		This study
UESC11028	*S. x-signatus*		Maracás, Bahia, Brazil	13°25'42"S, 40°26'16"W	OK161178	OK157307	This study
UESC11029	*S. x-signatus*		Maracás, Bahia, Brazil	13°25'42"S, 40°26'16"W	OK161179	OK157308	This study
UESC11030	*S. x-signatus*		Maracás, Bahia, Brazil	13°25'42"S, 40°26'16"W	OK161180	OK157309	This study
UESC11031	*S. x-signatus*		Maracás, Bahia, Brazil	13°25'42"S, 40°26'16"W	OK161181	OK157310	This study
UESC11032	*S. x-signatus*		Maracás, Bahia, Brazil	13°25'42"S, 40°26'16"W	OK161190	OK157311	This study
UESC11040	*S. x-signatus*		Serrinha, Bahia, Brazil	11°37'28"S, 38°58'26"W	OK161191	OK157318	This study
UESC11041	*S. x-signatus*		Serrinha, Bahia, Brazil	11°37'28"S, 38°58'26"W	OK161192		This study
UESC11042	*S. x-signatus*		Serrinha, Bahia, Brazil	11°37'28"S, 38°58'26"W	OK161193	OK157319	This study
UESC11043	*S. x-signatus*		Serrinha, Bahia, Brazil	11°37'28"S, 38°58'26"W	OK161194	OK157320	This study
UESC11044	*S. x-signatus*		Serrinha, Bahia, Brazil	11°37'28"S, 38°58'26"W	OK161195	OK157321	This study
UESC11136	*S. x-signatus*		Nazaré, Bahia, Brazil	13°2'8"S, 39°0'12"W	OK161196	OK157322	This study
UESC11045	*S. x-signatus*		Jequié, Bahia, Brazil	13°51'4"S, 40°4'52"W	OK161197	OK157323	This study

Voucher	Specie	Type locality	Location sampled	Geografic Coordinates	Number of acess in GenBank		Reference
					16S	COI	
UESC11046	*S. x-signatus*		Paulo Afonso, Bahia, Brazil	9°24′39″S, 38°14′9″W	OK161174	OK157324	This study
UESC11057	*S. x-signatus*		Jequié, Bahia, Brazil	13°51′4″S, 40°4′52″W	OK161199	OK157325	This study
UESC11065	*S. x-signatus*		Guanambi, Bahia, Brazil	14°12′26″S, 42°46′55″W	OK161200	OK157326	This study
UESC11066	*S. x-signatus*		Guanambi, Bahia, Brazil	14°12′26″S, 42°46′55″W	OK161201	OK157327	This study
UESC11067	*S. x-signatus*		Guanambi, Bahia, Brazil	14°12′26″S, 42°46′55″W	OK161202	OK157328	This study
UESC11069	*S. x-signatus*		Guanambi, Bahia, Brazil	14°12′26″S, 42°46′55″W	OK161203	OK157329	This study
UESC11070	*S. x-signatus*		Guanambi, Bahia, Brazil	14°12′26″S, 42°46′55″W	OK161204	OK157330	This study
UESC11071	*S. x-signatus*		Guanambi, Bahia, Brazil	14°12′26″S, 42°46′55″W	OK161205	OK157331	This study
UESC11072	*S. x-signatus*		Guanambi, Bahia, Brazil	14°12′26″S, 42°46′55″W	OK161206	OK157332	This study
UESC11073	*S. x-signatus*		Guanambi, Bahia, Brazil	14°12′26″S, 42°46′55″W	OK161207	OK157333	This study
UESC11074	*S. x-signatus*		Guanambi, Bahia, Brazil	14°12′26″S, 42°46′55″W	OK161208	OK157334	This study
UESC11075	*S. x-signatus*		Jequié, Bahia, Brazil	13°51′4″S, 40°4′52″W	OK161209	OK157335	This study
UESC11076	*S. x-signatus*		Jequié, Bahia, Brazil	13°51′4″S, 40°4′52″W	OK161210	OK157336	This study
307*	*S. x-signatus*		Ilhéus, Bahia, Brazil	14°47′50″S, 39°2′8″W	OK161177	OK157354	This study
525*	*S. x-signatus*		Nazaré, Bahia, Brazil	13°2′8″S, 39°0′12″W	OK161175	OK157337	This study
526*	*S. x-signatus*		Nazaré, Bahia, Brazil	13°2′8″S, 39°0′12″W	OK161176	OK157338	This study
UESC11011	*S. x-signatus*		Jequié, Bahia, Brazil	13°51′4″S, 40°4′52″W	OK161183		This study
UESC11046	*S. x-signatus*		Jequié, Bahia, Brazil	13°51′4″S, 40°4′52″W	OK161198		This study

* City was not informed, ** data is not reported in GenBank.
